# Supplementary figures and images for: Comparative Neuroanatomy of Pediveliger Larvae of Various Bivalves from the Sea of Japan
Source: Biology (Basel). 2023 Oct 17;12(10):1341. doi: 10.3390/biology12101341 (PMC10604817; doi:10.3390/biology12101341)

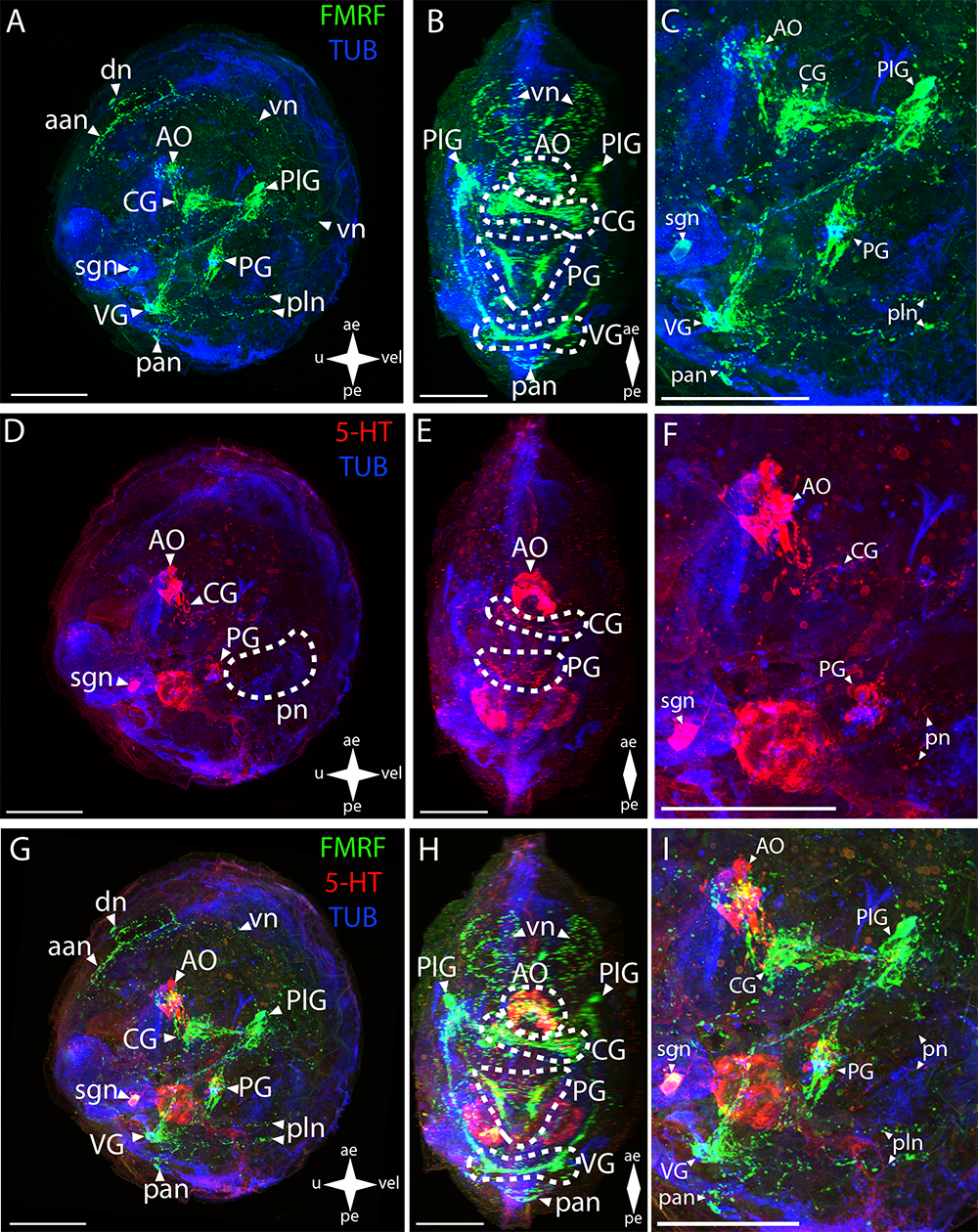

Supplement: Supplementary file 1 [file biology-12-01341-s001.zip › Figure S1.tif]

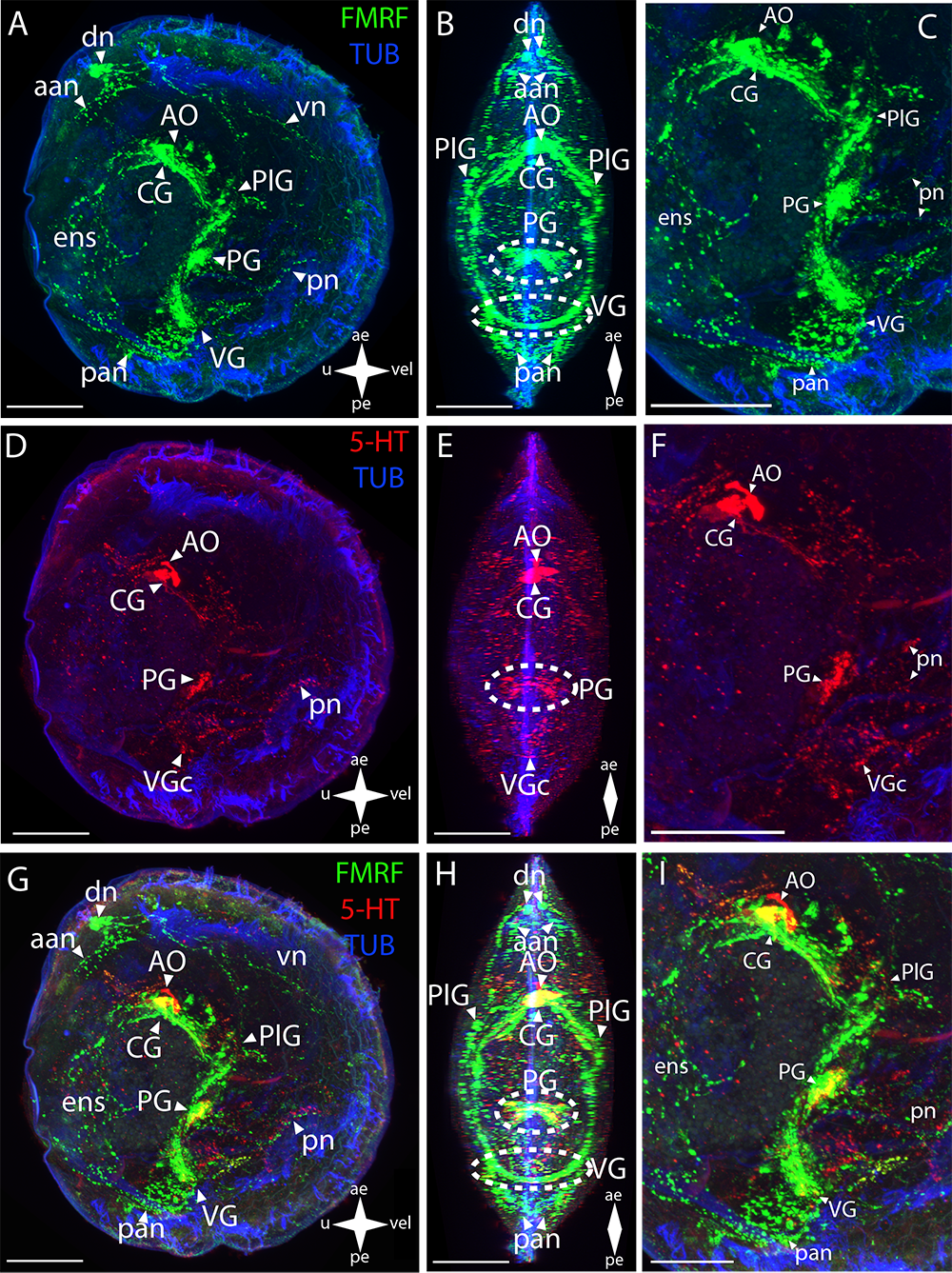

Supplement: Supplementary file 1 [file biology-12-01341-s001.zip › Figure S2.tif]
